# Supplementary material for: Conformal TiO2 Aerogel-Like Films by Plasma Deposition: from Omniphobic Antireflective Coatings to Perovskite Solar Cell Photoelectrodes
Source: ACS Appl Mater Interfaces. 2024 Jul 20;16(30):39745–60. doi: 10.1021/acsami.4c00555 (PMC11299147; doi:10.1021/acsami.4c00555)
Supplement: Supplementary file 1 — am4c00555_si_001.pdf [file am4c00555_si_001.pdf]

Supplementary Information

Conformal TiO_2 aerogel-like films by plasma deposition: from omniphobic antireflective coatings to perovskite solar cells photoelectrodes

Jose M. Obrero[a], Lidia Contreras-Bernal[a], Francisco J. Aparicio Rebollo,[a,b] Teresa C. Rojas[a], Francisco J. Ferrer,[c] Noe Orozco,[a] Zineb Saghi,[d] Triana Czermak,[a] Jose M. Pedrosa,[e] Carmen López-Santos,[a,b] Kostya (Ken) Ostrikov,[f] Ana Borras,[a] Juan Ramón Sánchez-Valencia,[a]* Angel Barranco.[a]*

a) Nanotechnology on Surfaces and Plasma Laboratory, Materials Science Institute of Seville (CSIC-US), C/ Américo Vespucio 49, 41092, Seville, Spain.

b) Departamento de Física Aplicada I, Escuela Politécnica Superior, Universidad de Sevilla, Spain. c/ Virgen de Africa 4101

c) Centro Nacional de Aceleradores (CNA, CSIC-Universidad de Sevilla)

d) Univ. Grenoble Alpes, CEA, LETI, F-38000 Grenoble, France

e) Departamento de Sistemas Físicos, Químicos y Naturales. Universidad Pablo de Olavide, Ctra. Utrera Km. 1, 41013 Sevilla, Spain.

f) School of Chemistry and Physics and Centre for Materials Science, Queensland University of Technology (QUT), Brisbane, QLD 4000, Australia.

corresponding authors: angel.barranco@csic.es, jrsanchez@icmse.csic.es

S1.- Characterization of the TiPC plasma polymer

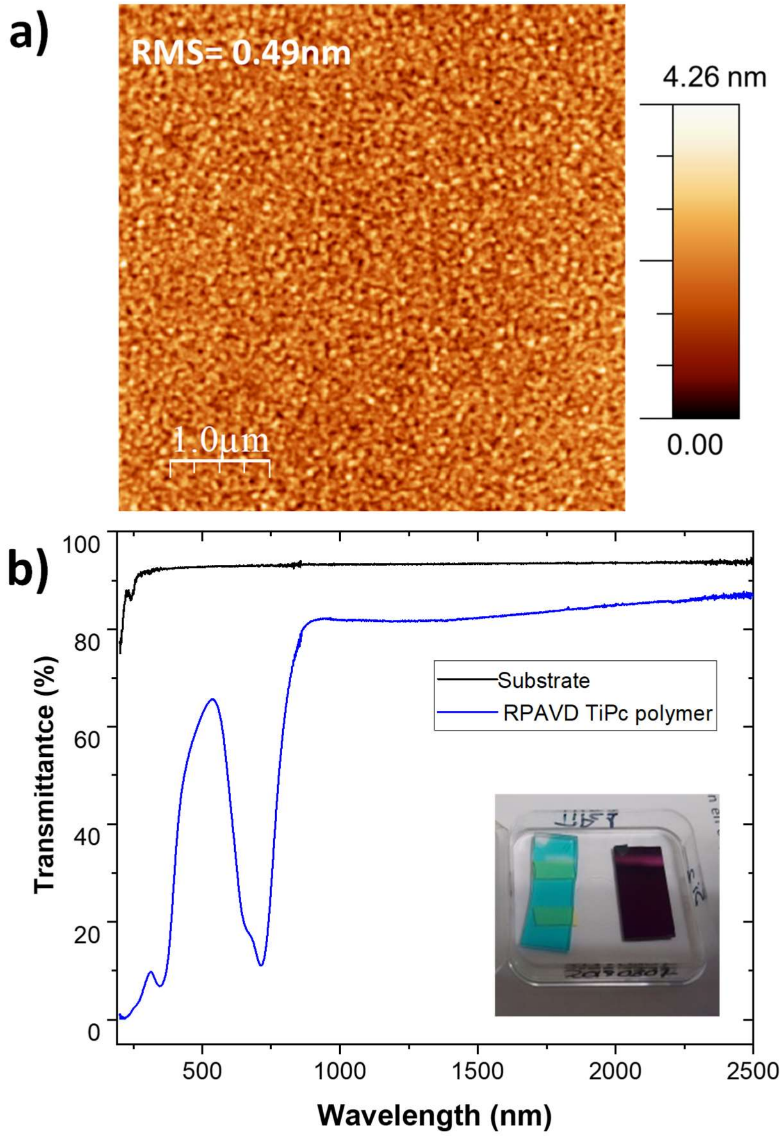

Figure S1.- Characterization of the TiPc plasma polymers. a) AFM micrograph of a TiPC film b) UV-VIS-NIR spectra of a RPAVD TiPc plasma polymer a as well as the bare fused silica substrate used. The inset show a picture of the TiPc plasma polymer deposited on fused silica and Si(100).

S2.- XPS analysis

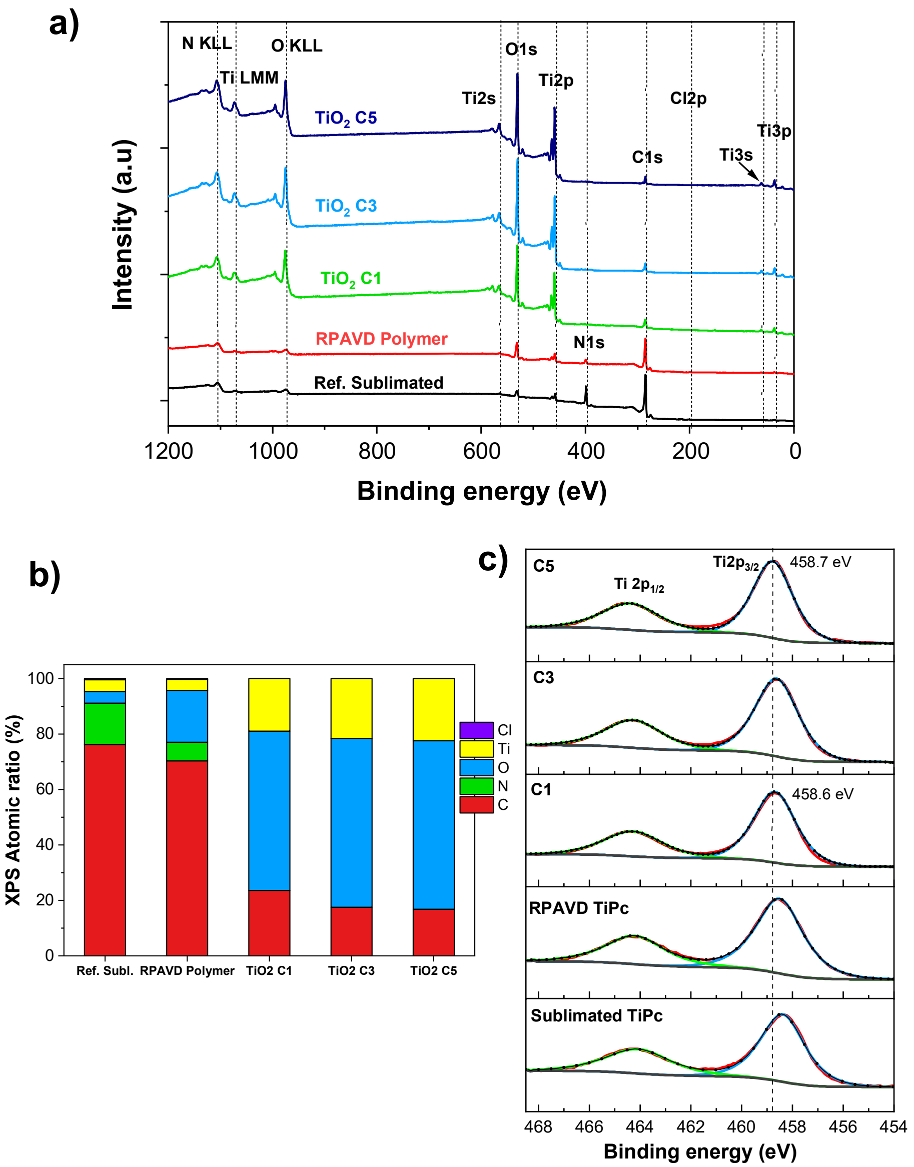

Figure S2. **XPS characterization** a) XPS survey spectra of sublimated, polymerized and three aerogel-like TiO_2 thin films as indicated. b) Relative atomic ratios deduced from the XPS analysis of the samples in a). c) XPS spectra of the Ti2p core level of the samples in a). The Ti2p3/2 binding energies of the porous TiO2 samples are in the range 450.7-458.6 characteristics of TiO_2 (NIST). Note that the TiPc plasma polymer and sublimated TiPc reference are slightly shifted to lower binding energies due to the different chemical environment of the cation although also corresponds to Ti(IV) core levels. All the XPS analyses correspond to as-deposited samples without any surface cleaning procedure.

Table S1.- XPS atomic percentages for the samples in Figure S2b).

	XPS Atomic Ratio (%)				
	**C**	**N**	**O**	**Ti**	**Cl**
**Sublimated (reference)**	76.2	15.0	4.1	4.3	0.4
**RPAVD polymer**	70.3	6.7	18.7	4.0	0.3
**TiO_2 C1**	23.6	0.0	57.5	18.9	0.0
**TiO_2 C3**	17.6	0.0	60.9	21.5	0.0
**TiO_2 C5**	16.8	0.0	60.7	22.4	0.0

S3.- FIB-SEM characterization

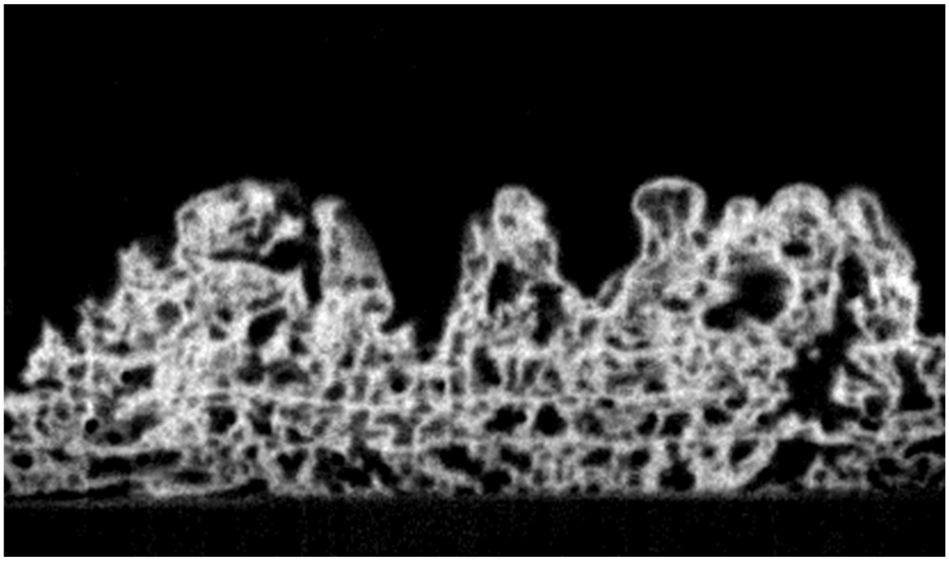

Figure S3 (Video S3).- **Aerogel-like film porous structure characterization**. FIB-SEM characterization of an aerogel-like film. Image taken from the video sequence of the FIB characterization of an aerogel-like TiO_2 thin film. The full video corresponds to the sample in Figure 3 (see main text). Video S3 is available as supplementary information material.

S4.- Plasma etching at RT

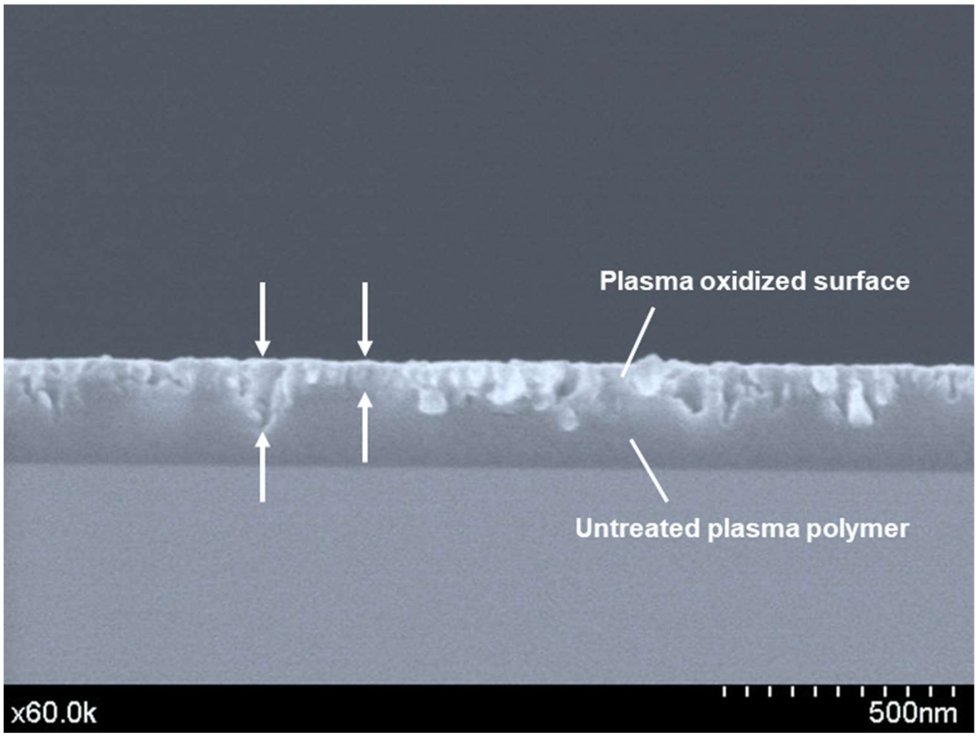

Figure S4.- Plasma etching treatment at room temperature. Cross-sectional SEM micrograph of a ~210 nm thick RPAVD TiPc plasma polymer subjected to an oxygen plasma etching treatment at room temperature as described in the experimental section. The image shows how the oxidation of the plasma polymer film is incomplete and restricted to a surface region of ~50-100 nm thick as indicated.

S5.- XRD

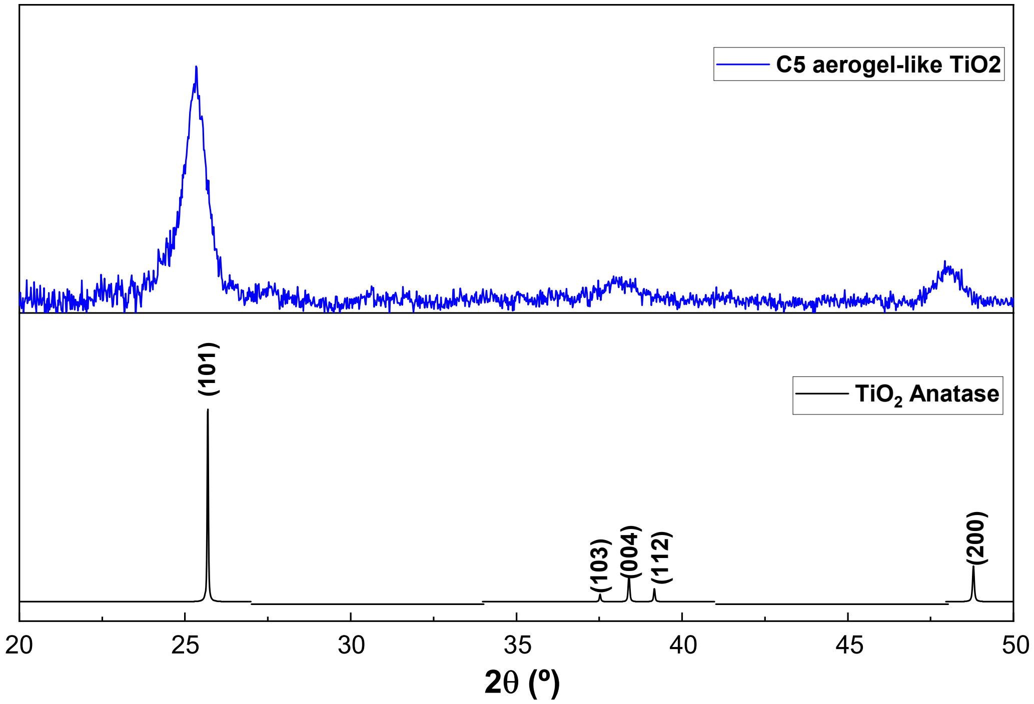

Figure S5.- XRD characterization of a partially crystalline aerogel-like thin film. Glancing angle XRD of a C5 of 640 nm thick aerogel-like TiO_2 thin film. Anatase phase diffraction peaks are included for comparison (catalog JCPDS-ICDD 2003 file number 78-2486)

S6.- Effective medium approximation.

We have used an effective medium approximation (EMA), concretely the Bruggeman model, which has been extensively used in porous systems for the refractive index determination,[1,2] to evaluate the effective refractive index of the ultraporous layers synthesized in this work. This model establishes a relationship between the material's effective refractive index (n_{eff}) and its porosity, assuming a homogeneous distribution of pores within the material.[3] By TEM we have observed a mixture of anatase crystals embedded in an amorphous matrix. However, determining the relative concentration between these two phases is not straightforward. We have assumed a mixture (50:50) of amorphous and anatase phases with n_{ox} (Amorp)= 2.38 (@735 nm) and n_{ox} (Anat)= 2.54 (@735 nm) and we have considered that the pores can be filled with ir (n_{air} = 1) or water (n_{water} = 1.33).[4] Since the TiO_2 layer has a specific porosity, P, the Bruggeman equation for these four media can be expressed as follows:

$$P(x)\cdot\frac{n_{water}^2-n_{eff}^2}{n_{water}^2+2n_{eff}^2}+P\cdot(1-x)\cdot\frac{n_{air}^2-n_{eff}^2}{n_{air}^2+2n_{eff}^2}+(1-P)\cdot 0.5\cdot\frac{n_{ox}^2(Amorp)-n_{eff}^2}{n_{ox}^2(Amorp)+2n_{eff}^2}+(1-P)\cdot 0.5\cdot\frac{n_{ox}^2(Anat)-n_{eff}^2}{n_{ox}^2(Anat)+2n_{eff}^2}=0$$

where x is the fraction of the pores filled with water, and the factor 0.5 is due to the assumption that the mixture is formed by 50% amorphous and 50% anatase TiO_2 medium.

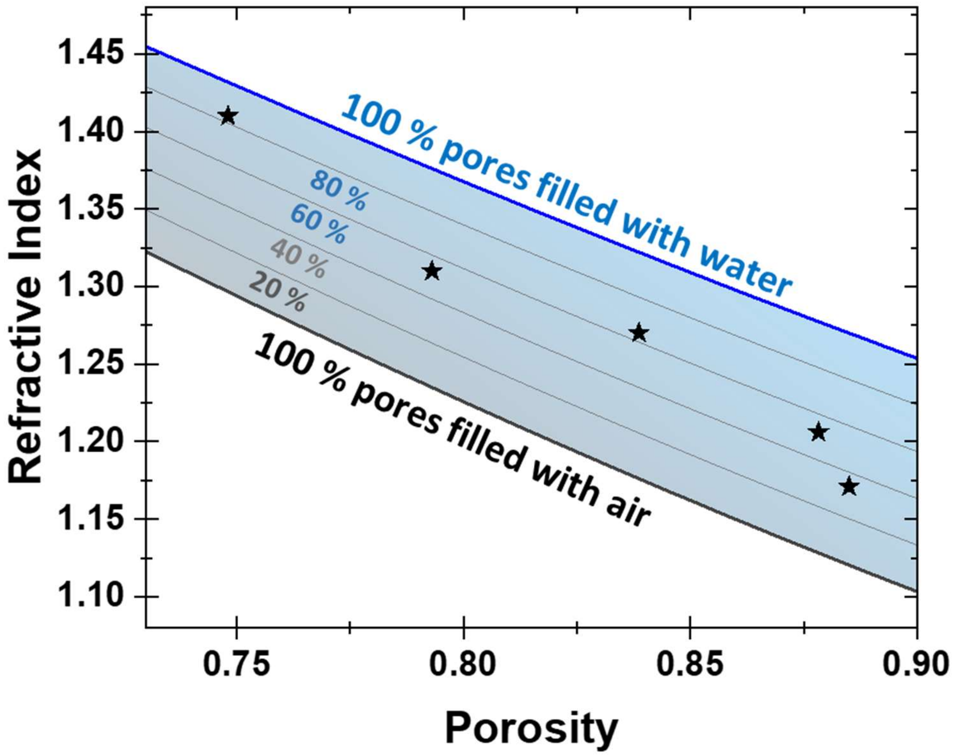

Figure S6.- Refractive index and porosity in aerogel-like films.- Refractive index versus porosity values for the films in Figure 8c). The lines on the graph represent the values obtained from the Bruggeman equation assuming a mixture (50:50) media of amorphous and anatase phases with n_{ox} (Amorp)= 2.38 (@735 nm) and n_{ox} (Anat)= 2.54 (@735 nm). It is considered that the

pores can be filled with ir (n_{air} = 1) or water (n_{water} = 1.33).[4], which vary according to the water percentage within the pores across the studied porosity range

This implicit equation is evaluated in Figure S6 for the pores filled with air (black curve) or water (blue curve), delimiting the lower and upper possible limits of the refractive index for each porosity value. It is worth mentioning that the partial filling of the pores with environmental moisture is a common effect for porous samples, especially for plasma-synthesized layers whose surface is highly hydrophilic due to hydroxylation. The hydrophilic behavior of the surface is gradually lost with time, and the recovery time to reach the hydrophobic nature of the TiO_2 surface depends on many factors, such as the aging period, environmental conditions (temperature and relative humidity), or dark storage, among others.
Figure S6 also displays the refractive index extracted from ellipsometry (Figure 8 c in the manuscript) versus the porosity determined by RBS (Figure 4 a in the manuscript) with stars. It can be noted that the refractive index values are in the expected range for the anatase:amorphous mixture case, and show values of percentage of pores filled with water of 80 and 35% for the C1 and C5 aerogel-like TiO_2.

1. Khardani, M., Bouaïcha, M. & Bessaïs, B. Bruggeman effective medium approach for modelling optical properties of porous silicon: comparison with experiment. *Phys. Status Solidi C* **4**, 1986–1990 (2007).

2. Sela, M. & Haspel, C. Predicting the refractive index of amorphous materials using the Bruggeman effective medium approximation. *Appl. Opt.* **59**, 8822 (2020).

3. Álvarez-Herrero, A., Heredero, R. L., Bernabeu, E. & Levy, D. Adsorption of water on porous Vycor glass studied by ellipsometry. *Appl Opt* **40**, 527–532 (2001).

4. Jolivet, A. *et al.* Structural, optical, and electrical properties of TiO2 thin films deposited by ALD: Impact of the substrate, the deposited thickness and the deposition temperature. *Appl. Surf. Sci.* **608**, 155214 (2023).

S7. Additional characterizations of annealed films

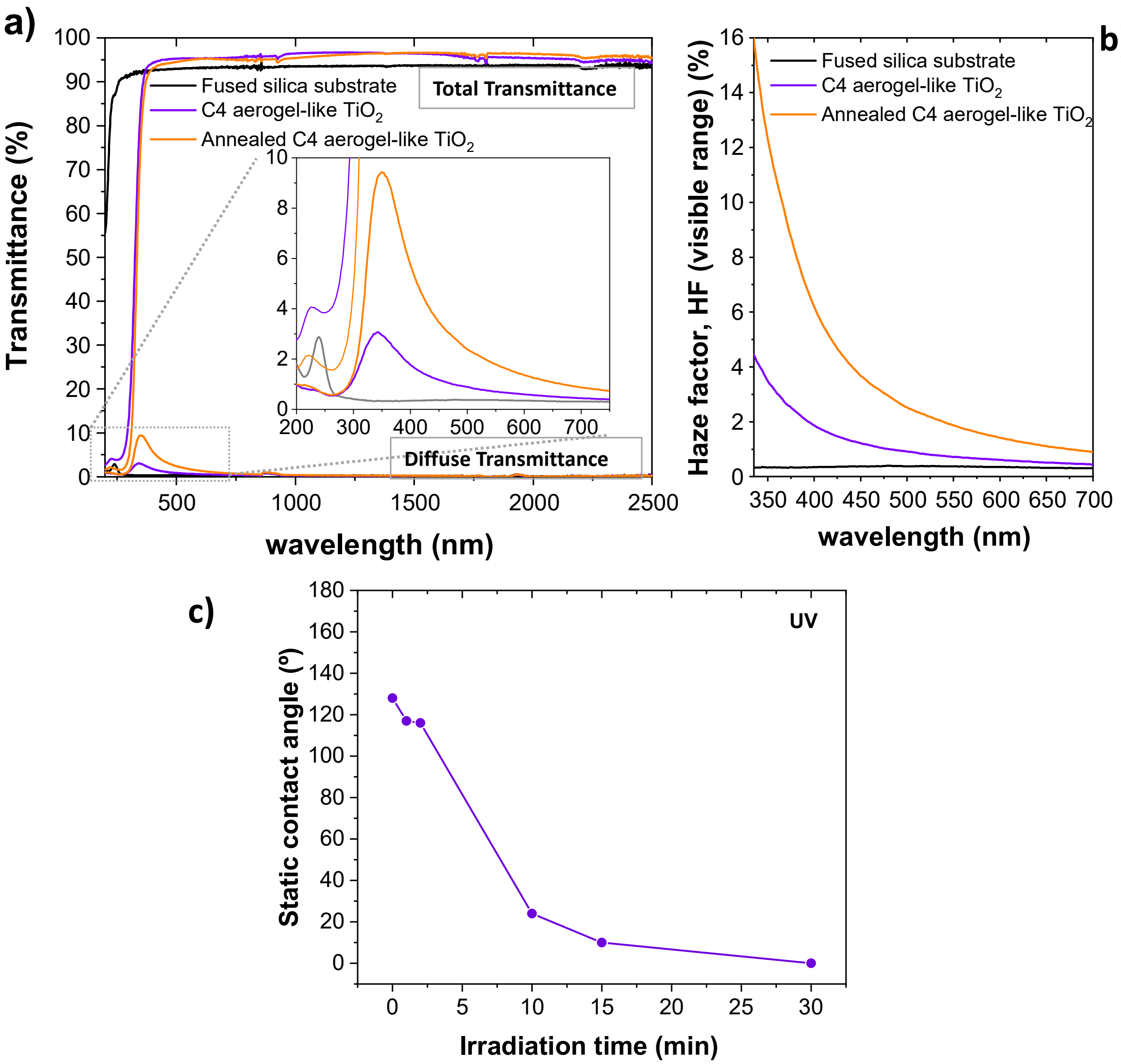

Figure S7.- Optical and photocatalytic characterization of annealed films. a) Total and diffuse transmittance spectra of a C4 aerogel-like film and the same film annealed. The inset shows the diffuse transmittance spectra enlarged b) Haze factor calculated from the spectra in a) c) Water contact angle evolution under UV irradiation of an annealed TiO_2 aerogel-like film.

The annealing provokes an increase in the diffuse transmittance, which is enhanced in the visible range and reaches 9.5% at 340 nm. We have calculated the Haze factor (HF) as: $HF = \frac{T_d}{T_T} \cdot 100$ (%) being T_T and T_d the total and diffuse transmittance values, respectively. It can be seen that the HF for the annealed C4 aerogel-like TiO_2 sample is higher than the as-prepared one, reflecting that the transformation to anatase provokes an increase in the diffuse light transmittance of the samples.

Table S2.- XPS atomic percentages of an annealed C4 film after and before the UV irradiation. A reference TiO_2 mesoporous anatase film is included for comparison.

	XPS Atomic Ratio (%)				
	C	N	O	Ti	Cl
mp-TiO_2 anatase (ref.)	14.0	0.0	60.7	25.3	0.0
TiO_2 C4 anatase	20.0	0.0	55.3	24.7	0.0
TiO_2 C4 anatase (UV)	17.3	0.0	61.4	21.3	0.0

S8.- Supplementary photovoltaic characterization.

Table S3). Photovoltaic parameters statistic of perovskite solar cells extracted from density current-voltage curves measured under 1 sun-AM 1.5G illumination in reverse scan and using a mask of 0.14 cm^2. The photovoltaic data of the champion cell are in brackets.

	J_{sc} ($mA \cdot cm^{-2}$)	V_{OC}(V)	FF (%)	EFF (%)
**Ref**	19.6 (19) ± 0.4	1.04 (1.03) ±0.01	75 (82) ± 4	15.4 (16) ±0.5
**C2**	18.1 (17.2) ± 1	1.02 (1.04) ± 0.02	71 (78) ± 6	13 (14.1) ± 1
**C3**	18.2 (18.5) ± 0.8	1.02 (1.04) ± 0.02	73 (75) ± 4	13.5 (14.4) ± 1.1
**C4**	18.3 (18.9) ± 0.8	1.04 (1.05) ± 0.01	75 (81) ±4	14.3 (16.1) ± 1.2
**C5**	18.4 (18.7)± 0.6	1.04 (1.05) ± 0.01	75 (79) ± 5	14.4 (15.5) ± 1

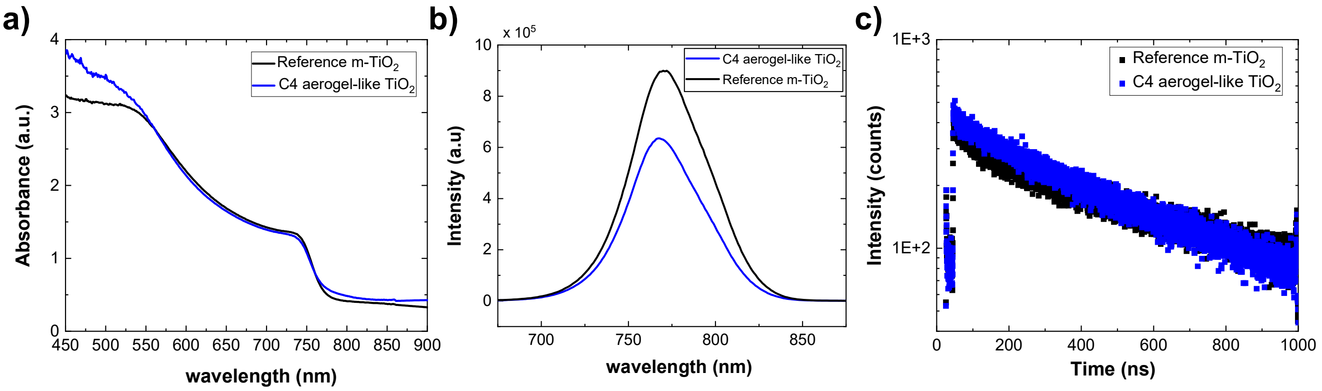

Figure S8. Optical and luminescence characterization. a) Absorbance spectra of perovskite films deposited on a commercial reference mesoporous TiO_2 and a C4 TiO_2 aerogel-like film. b) Steady-state luminescence (divided by the absorbance at the excitation wavelength) of the films. c) Time-resolved PL of the films. The excitation wavelength for the PL measurements was 490 nm.

Table S4). Biexponential fitting of the decay curves shown in Figure S8, according to the formula: $A_1 \cdot e^{-\frac{t}{\tau_1}} + A_2 \cdot e^{-\frac{t}{\tau_2}}$. The table also includes the associated relative amplitude (Rel) and the average lifetime ($\langle\tau\rangle$)

ETL	τ_1(ns)	A_1(ns)	Rel_1(%)	τ_2(ns)	A_2(ns)	Rel_2(%)	$\langle\tau\rangle$ (ns)
m-TiO2/perovskite	37.3	42.6	1.19	513.4	256.2	98.81	507.8
aerogel–like TiO_2/perovskite	41.5	11.9	0.35	446.4	316.2	99.65	444.9

S9. Synthesis of porous oxide films using metal-containing precursors.

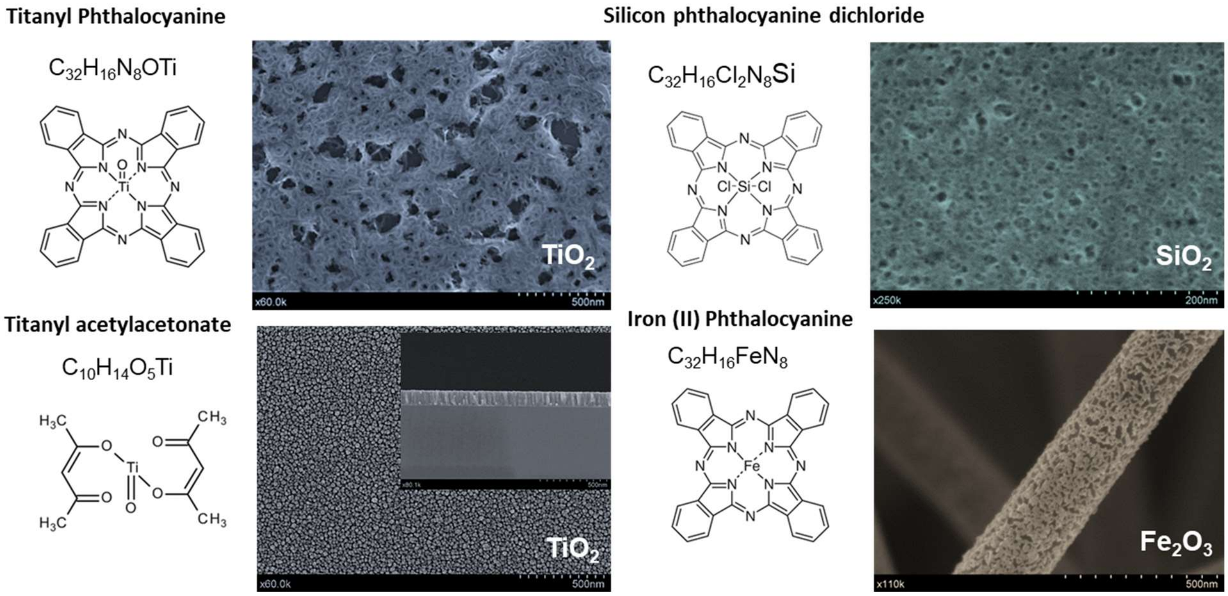

Figure S9.- **Generalization of the aerogel-like thin film synthetic procedure**. Examples of porous films obtained through the sequence of plasma polymerization and plasma etching described in the text but utilizing different metal-containing precursors. The three phthalocyanine precursors yield highly porous films, displaying distinct similarities to the aerogel films discussed in the text. However, the polymerization and plasma oxidation of titanyl acetylacetonate result in a more compact and denser microstructure characterized by packed nanocolumns. A comprehensive investigation of the properties of these films falls beyond the scope of this study.
